# Implications of C1q/TNF-related protein-3 (CTRP-3) and progranulin in patients with acute coronary syndrome and stable angina pectoris

**DOI:** 10.1186/1475-2840-13-14

**Published:** 2014-01-13

**Authors:** Kyung Mook Choi, Soon Young Hwang, Ho Chel Hong, Hae Yoon Choi, Hye Jin Yoo, Byung-Soo Youn, Sei Hyun Baik, Hong Seog Seo

**Affiliations:** 1 Division of Endocrinology and Metabolism, Department of Internal Medicine, College of Medicine, Korea University, Seoul, Korea; 2 Department of Biostatistics, College of Medicine, Korea University, Seoul, Korea; 3 AdipoGen, Inc, Venture Building B, Songdo Technopark, 7-50 Songdo-dong, Yeonsu-gu, Incheon, Korea; 4 Division of Cardiology, Department of Internal Medicine, Korea University Guro Hospital, 80 Guro-Dong, Guro-Gu, Seoul 152-050, Korea

**Keywords:** C1q/tumor necrosis factor-related protein-3, Progranulin, Coronary artery disease, Acute coronary syndrome, Stable angina pectoris, Adipokines

## Abstract

**Background:**

C1q/TNF-related protein-3 (CTRP-3), an adiponectin paralog, and progranulin were recently identified as novel adipokines which may link obesity with glucose dysregulation and subclinical inflammation. We analyzed the relationship between CTRP-3, progranulin and coronary artery disease (CAD) in Korean men and women.

**Methods:**

Circulating CTRP-3 and progranulin levels were examined in 362 Korean adults with acute coronary syndrome (ACS, n = 69), stable angina pectoris (SAP, n = 85), and control subjects (n = 208) along with various kinds of cardiometabolic risk factors.

**Results:**

CTRP-3 concentrations were significantly decreased in patients with ACS or SAP compared to control subjects (*P* <0.001, respectively), whereas progranulin and adiponectin levels were similar. Correlation analysis adjusted for age and gender exhibited that CTRP-3 levels showed significant negative relationship with glucose (r = -0.110, *P* = 0.041) and high sensitive C-reactive protein (hsCRP) levels (r = -0.159, *P* = 0.005), and positive relationship with HDL-cholesterol (r = 0.122, *P* = 0.025) and adiponectin levels (r = 0.194, *P* <0.001). In a multivariate logistic regression analysis, the odds ratio for CAD risk was 5.14 (95% CI, 1.83-14.42) in the second, and 9.04 (95% CI, 2.81-29.14) in the first tertile of CTRP-3 levels compared to third tertile after adjusting for other cardiometabolic risk variables.

**Conclusions:**

Patients with ACS or SAP had significantly lower circulating CTRP-3 concentrations compared to control subjects, although progranulin levels were not different. These results suggest the possibility that CTRP-3 might be useful for assessing the risk of CAD.

**Trial registration:**

(Clinical trials No.: NCT01594710)

## Background

Visceral obesity is the one of the major causes of cardiovascular morbidity and mortality in industrialized countries [1]. Adipose tissue secretes various kinds of bioactive molecules termed adipokines which contribute to the development of obesity-related disorders including cardiovascular disease (CVD). Adiponectin is the insulin-sensitizing adipokine with anti-inflammatory and anti-atherogenic properties [2]. Hypoadiponectinemia has been associated with an increased risk of coronary artery disease (CAD) in several though not all studies [3].

Recently, a new family of secreted proteins, C1q/TNF-related protein (CTRP), was cloned on the basis of sequence homology with adiponectin [4]. This family of adiponectin paralogs might have pivotal implications in energy homeostasis and obesity-related inflammation [5]. Each CTRP family member has its own unique tissue expression profile and function in regulating glucose and/or fat metabolism [6]. Recent studies have shown that CTRP-3 (synonyms CORS-26, cartducin and cartonectin) is a potent anti-inflammatory adipokine that inhibits proinflammatory pathways in monocytes and adipocytes [7,8]. CTRP-3 administration significantly lowers glucose levels, but does not influence the levels of insulin or adiponectin in normal and insulin-resistant *ob/ob* mice [9]. Using a recently developed enzyme-linked immunosorbent assay (ELISA), we reported that circulating CTRP-3 levels were elevated in patients with glucose metabolism dysregulation [10]. In non-diabetic subjects, we observed that CTRP-3 concentrations exhibit a significant negative correlation with cardiometabolic risk factors and positive correlation with adiponectin levels [11]. In oligonucleotide arrays comparing expression profiling of injured and control artery, CTRP-3 is found in rat carotid artery following balloon angioplasty [12]. Moreover, Maeda et al. reported that CTRP-3 has a novel biological role in promoting vascular smooth muscle cell proliferation in blood vessel wall after injury [13]. On the other hand, Yi et al. revealed that CTRP-3 is a novel antiapoptotic, proangiogenic and cardioprotective adipokine, the expression of which is significantly inhibited after MI [14].

Recently, progranulin was identified as a key adipokine mediating high fat diet-induced insulin resistance and obesity through interleukin-6 (IL-6) in adipose tissue [15]. We previously reported that progranulin levels were significantly higher in individuals with type 2 diabetes and were associated with macrophage infiltration in omental adipose tissue [16]. Circulating progranulin levels was an independent predictor for increased carotid intima-media thickness in subjects without metabolic syndrome, but not in those with metabolic syndrome [11]. Kojima et al. found progranulin expression in advanced human atherosclerotic plaque [17]. Moreover, the expression of progranulin reduces inflammation and its degradation into granulins peptides enhances inflammation in atherosclerotic plaque, which may contribute to the progression of atherosclerosis [17]. However, to the best of author’s knowledge, there has been no previous report on the implication of CTRP-3 or progranulin in humans with cardiovascular disease (CAD).

In the present study, we compared circulating CTRP-3 and progranulin levels in patients with CAD and investigated whether CTRP-3 or progranulin is significantly associated with CAD prevalence after adjustment for well-known CAD risk factors.

## Methods

### Study participants and definition of coronary artery disease

Acute coronary syndrome (ACS) patients, who were admitted to the coronary care units of the division of cardiology at Guro hospital in Korea University Medical Center between March 2011 and 31 December 2012, were consecutively recruited for this study. Among them, acute myocardial infarction (AMI) was defined as a typical increase and gradual decrease of biochemical markers of myocardial necrosis (detection of a rise and/or fall of cardiac biomarker values such as CK-MB and/or troponin-T with at least one value above the 99^th^ percentile upper reference limit) and at least one of the following: ischemic symptoms, electrocardiogram (ECG) changes indicative of new ischemia (new ST-T changes or new left bundle branch block [LBBB]), development of pathologic Q waves on ECG, and imaging evidence of new loss of viable myocardium or new regional wall motion abnormality [18]. The criteria for unstable angina included symptoms of angina at rest, a new-onset exertional angina, or a recent acceleration of angina. In case of stable angina pectoris (SAP), the symptom should have been stable for at least for 6 months and ≥50% luminal narrowing in at least one major coronary artery was confirmed on coronary angiography. Control subjects were recruited from the participants for a routine health check-up in the Health Promotion Center of Korea University Guro Hospital between March 2012 and December 2012. For control group, we exclude the participants had a history of CVD (myocardial infarction, unstable angina, stroke, or cardiovascular revascularization), type 2 diabetes, stage 2 hypertension (resting blood pressure, ≥160/100 mmHg), malignancy, or severe renal or hepatic disease. All participants provided written informed consent and Korea University Institutional Review Board, in accordance with the Declaration of Helsinki of the World Medical Association, approved this study protocol.

### Anthropometric and laboratory measurements

BMI was calculated as weight/height^2^ (kg/m^2^) and waist circumference was measured at the midpoint between the lower border of the rib cage and the iliac crest. All blood samples were obtained the morning after a 12-hour overnight fast, and were immediately stored at -80°C for subsequent assays. Serum triglyceride and high density lipoprotein cholesterol (HDL-C) levels were determined enzymatically using a model 747 chemistry analyzer (Hitachi, Tokyo, Japan). The glucose oxidase method was used to measure plasma glucose levels and an electrochemiluminescence immunoassay (Roche Diagnostics, Indianapolis, IN, USA) was used to measure insulin levels. Estimated glomerular filtration rate (eGFR) was calculated from the Modification of Diet in Renal Disease (MDRD) study equation: (ml/min/1.73 m^2^) = 175 X (Scr)^-1.154^ X (Age)^-0.203^ X (0.742 if female) [19]. Latex-enhanced turbidimetric immunoassay (HiSens hsCRP LTIA; HBI, Anyang, Korea) was used for measurement of hsCRP. Adiponectin levels were measured by ELISA (AdipoGen, Incheon, Korea). Newly-developed ELISA was used for measurement of CTRP-3 (AdipoGen, Incheon, Korea; intra- and inter-assay CVs: 7.3 ± 1.0% and 5.8 ± 2.7%, respectively) and progranulin (AdipoGen, Incheon, Korea; intra- and inter-assay CVs: 5.8 ± 0.6% and 7.0 ± 0.3%, respectively) levels.

### Statistical analyses

Data are presented as mean ± SD or median (interquartile range). We performed the Shapiro-Wilk test to evaluate normality. Differences between groups were tested using a one-way ANOVA test or the Kruskal-Wallis test for continuous variables. Tukey’s multiple comparison test and the Wilcoxon rank-sum test were used for multiple comparisons. Bonferroni’s correction was used to adjust for multiple comparisons. The Chi-square test was used to test for differences in the distribution of categorical variables. Spearman’s partial correlation test was performed to determine the relationships between the CTRP-3 or progranulin or adiponectin and other variables. Multiple logistic regression analysis with CAD as a dependent variable was performed to identify the risk factors. We used SAS 9.2 (SAS institute, Cary, NC) and a P-value less than 0.05 was considered statistically significant.

## Results

### Baseline characteristic of subjects with or without CAD

**Figure 1:**
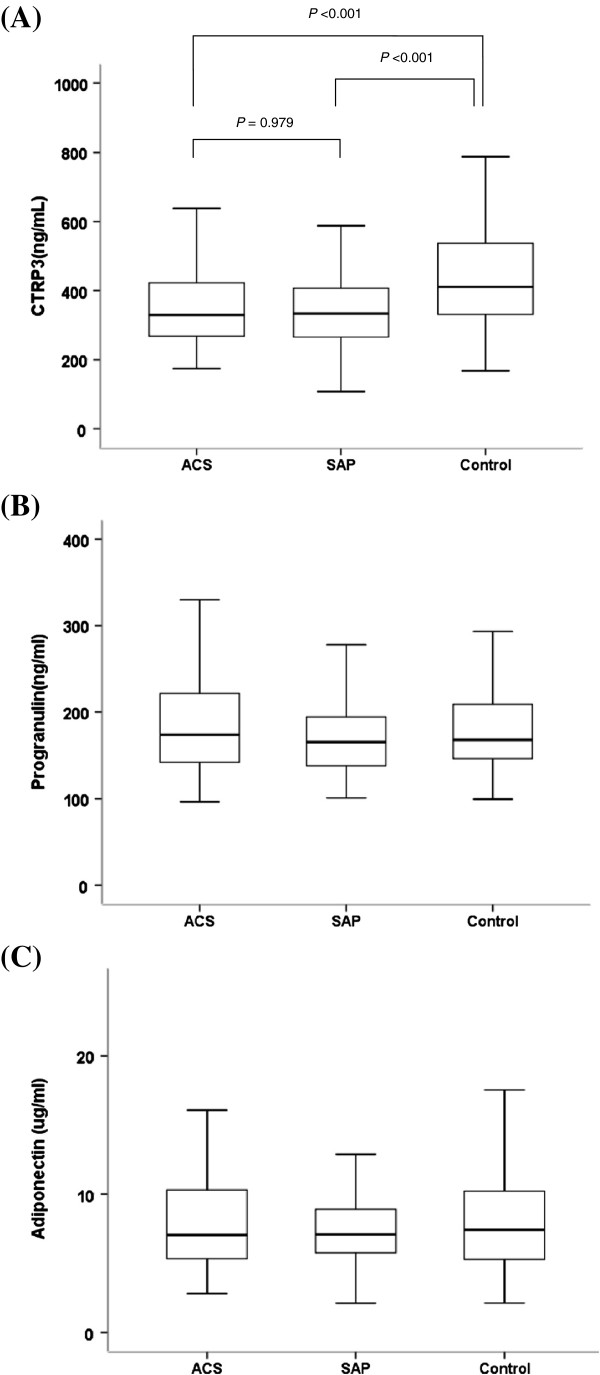
Difference of CTRP-3 (A), progranulin (B) and adiponectin (C) concentrations between patients with acute coronary syndrome (ACS) and stable angina pectoris (SAP) and control subjects.

The clinical and biochemical characteristics of the study subjects are presented in Table 1. Subjects with ACS or SAP showed significantly different age, waist circumference, diastolic blood pressure, total cholesterol, HDL-cholesterol, glucose and eGFR levels compared to control subjects. However, body weight, BMI, LDL-cholesterol and triglyceride levels were not different. In particular, subjects with CAD (ACS plus SAP) had increased levels of hsCRP, whereas adiponectin levels were not significantly different in this study. Interestingly, CTRP-3 concentrations were significantly decreased in patients with ACS or SAP compared to control subjects (*P* <0.001, respectively), although progranulin levels were similar (Figure 1).

**Table 1:** Baseline characteristics of the study participants

	**ACS**	**SAP**	**Control**	***P***
	**(n = 69)**	**(n = 85)**	**(n = 208)**	
Gender (M:F)	49:20	58:27	137:71	0.719
Age (years)	62 (54, 71)^a^	65 (59, 72)^a^	58 (55, 63)^b^	<0.001
Weight (kg)	67.6 ± 12.2	65.1 ± 10.9	64.8 ± 10.5	0.175
Body mass index (kg/m^2^)	24.6 (22.2, 26.7)	24.2 (22.5, 25.5)	24.1 (22.3, 26.0)	0.813
Waist circumference (cm)	87.8 ± 9.0^a^	86.4 ± 8.7^a^	82.7 ± 8.3^b^	<0.001
SBP (mmHg)	118 (106, 126)^a^	118 (110, 128)^a,b^	122 (111, 133)^b^	0.088
DBP (mmHg)	70 (62, 79)^a^	71 (64, 76)^a^	81 (72, 87.5)^a^	<0.001
Total cholesterol (mmol/l)	4.5 (3.5, 5.2)^a^	4.0 (3.5, 4.6)^a^	5.0 (4.3, 5.7)^b^	<0.001
LDL-cholesterol (mmol/l)	3.2 (2.6, 3.5)	2.9 (2.4, 3.8)	3.0 (2.4, 3.7)	0.782
Triglyceride (mmol/l)	1.2 (0.8, 1.8)	1.3 (0.8, 2.0)	1.3 (0.9, 1.8)	0.447
HDL-cholesterol (mmol/l)	1.1 (1.0, 1.3)^a^	1.2 (1.0, 1.4)^a^	1.4 (1.2, 1.6)^b^	<0.001
Glucose (mmol/l)	5.9 (5.4, 6.9)^a^	5.9 (5.3, 6.9)^a^	5.5 (5.2, 6.0)^a^	<0.001
Creatinine (mg/dl)	0.8 (0.6, 0.9)^a,b^	0.8 (0.6, 0.8)^a^	0.8 (0.7, 0.9)^b^	0.065
eGFR (mL/min/1.73 m^2^)	101.9 (85.1, 118.9)^a^	99.7 (85.1, 113.5)^a^	92.6 (84.2, 102.8)^b^	0.003
hsCRP (mg/dl)	1.3 (0.4, 8.6)^a^	0.9 (0.4, 1.3)^a^	0.3 (0.1, 0.9)^b^	<0.001
CTRP-3 (ng/ml)	329.6 (267.7, 424.1)^a^	332.9 (265.9, 407.3)^a^	410.6 (330.7, 537.2)^b^	<0.001
Progranulin (ng/ml)	173.8 (142.1, 221.6)	165.6 (138.2, 194.3)	168.3 (146.4, 209.1)	0.394
Adiponectin (μg/ml)	7.0 (5.3, 10.3)	7.1 (5.8, 8.9)	7.4 (5.3, 10.2)	0.875

Data are expressed as mean ± standard deviation or median (inter-quartile range).

a, b Same letters indicate no statistical significance based on Tukey’s HSD post-hoc test or Wilcoxon’s rank-sum test. *ACS*, acute coronary syndrome; *SAP*, stable angina pectoris; *SBP*, systolic blood pressure; *DBP*, diastolic blood pressure; *LDL*, low-density lipoprotein; *HDL*, high-density lipoprotein; *eGFR*, estimated glomerular filtration rate; *hsCRP*, high-sensitivity C-reactive protein; *CTRP-3*, C1q/TNF-related Protein-3.

### Correlation of circulating CTRP-3, progranulin and adiponectin concentrations with cardiometabolic risk factors adjusted for age and gender

Spearman correlation analysis adjusted for age and gender exhibited that CTRP-3 concentrations showed significant negative relationship with glucose (r = -0.110, *P* = 0.041) and hsCRP levels (r = -0.159, *P* = 0.005), and positive relationship with HDL-cholesterol (r = 0.122, *P* = 0.025) and adiponectin levels (r = 0.194, *P* <0.001) (Table 2). As expected, close relationship between adiponectin and various kinds of cardiometabolic risk factors, including waist circumference, triglyceride, HDL-cholesterol, glucose and hsCRP, was observed. On the other hand, circulating progranulin levels only had a negative correlation with HDL-cholesterol (r = -0.105, *P* = 0.048).

**Table 2:** Spearman correlation of circulating CTRP-3, progranulin and adiponectin levels with cardiometabolic risk factors adjusted for age and gender

	**CTRP-3**		**Progranulin**		**Adiponectin**	
	***r***	***P***	***r***	***P***	***r***	***P***
Weight	-0.007	0.898	0.028	0.592	-0.221	<0.001
Body mass index	0.001	0.979	0.028	0.593	-0.286	<0.001
Waist circumference	-0.068	0.243	0.058	0.311	-0.238	<0.001
Systolic blood pressure	-0.029	0.597	-0.073	0.168	-0.045	0.393
Diastolic blood pressure	0.097	0.073	-0.013	0.800	0.007	0.899
Total cholesterol	0.007	0.899	-0.083	0.121	0.102	0.056
LDL-cholesterol	-0.053	0.341	-0.041	0.453	0.065	0.233
Triglyceride	-0.097	0.076	0.081	0.129	-0.262	<0.001
HDL-cholesterol	0.122	0.025	-0.105	0.048	0.293	<0.001
Glucose	-0.110	0.041	-0.013	0.811	-0.220	<0.001
eGFR	-0.076	0.156	-0.060	0.271	0.039	0.458
Creatinine	0.072	0.178	0.075	0.157	-0.027	0.614
hsCRP	-0.159	0.005	0.061	0.271	-0.183	0.001
CTRP-3	-	-	0.091	0.091	0.194	<0.001
Progranulin	0.091	0.091	-	-	0.044	0.408
Adiponectin	0.194	<0.001	0.044	0.408	-	-

a, b, c Same letters indicate no statistical significance based on Tukey’s HSD post-hoc test or Wilcoxon’s rank-sum test. *LDL*, low-density lipoprotein; *HDL*, high-density lipoprotein; *eGFR*, estimated glomerular filtration rate; *hsCRP*, high-sensitivity C-reactive protein.

### Clinical and laboratory variables according to CTRP-3 level tertiles

Cardiometabolic risk factors according to CTRP-3 level tertiles for all study subjects are presented in Table 3. We found that lower CTRP-3 levels were associated with a higher weight, waist circumference and glucose levels. Moreover, lower HDL-cholesterol and adiponectin levels were observed in subjects with lower tertile of CTRP-3 levels.

**Table 3:** Clinical and laboratory variables according to CTRP-3 level tertiles

	**First tertile**	**Second tertile**	**Third tertile**	***P***
CTRP-3 (ng/ml)	267.7 (239.6, 291.5)^a^	367.8 (347.0, 401.0)^b^	553.8 (477.9, 634.9)^c^	<0.001
Age (years)	59 (54, 67)^a^	58 (55, 68)^a^	61 (58, 69)^b^	0.019
Weight (kg)	67.8 ± 9.6^a^	66.3 ± 11.7^a^	63.2 ± 10.7^b^	0.004
BMI (kg/m^2^)	24.4 ± 2.6	24.6 ± 3.0	24.0 ± 3.0	0.350
Waist circumference (cm)	85.9 ± 7.7^a^	85.2 ± 8.2^a^	82.5 ± 9.1^b^	0.011
SBP (mmHg)	119 (112, 128)	123 (109.5, 134)	120.5 (109.5, 132)	0.570
DBP (mmHg)	75.4 ± 12.0	78.2 ± 12.2	75.4 ± 11.1	0.110
Total cholesterol (mmol/l)	4.7 ± 1.1	4.6 ± 1.0	4.7 ± 1.0	0.616
LDL-cholesterol (mmol/l)	3.2 ± 0.9	2.9 ± 0.9	3.1 ± 0.8	0.111
Triglyceride (mmol/l)	1.3 (0.9, 2.0)	1.3 (0.9, 2.0)	1.2 (0.9, 1.6)	0.132
HDL-cholesterol (mmol/l)	1.2 (1.0, 1.5)^a^	1.2 (1.1, 1.4)^a^	1.3 (1.2, 1.6)^b^	0.001
Glucose (mmol/l)	5.7 (5.3, 6.4)^a,b^	5.8 (5.3, 6.5)^a^	5.5 (5.1, 6.0)^b^	0.029
Creatinine (mg/dl)	0.8 (0.7, 0.9)	0.8 (0.6, 0.9)	0.8 (0.7, 0.9)	0.080
eGFR (mL/min/1.73 m^2^)	96.2 (84.1, 110.1)	97.9 (84.8, 110.7)	91.5 (82.0, 107.4)	0.127
hsCRP (mg/dl)	0.6 (0.2, 1.4)	0.7 (0.1, 1.4)	0.5 (0.1, 0.9)	0.157
Progranulin (ng/ml)	165.0 (135.9, 191.1)	169.4 (144.0, 213.2)	171.4 (148.8, 210.6)	0.116
Adiponectin (μg/ml)	6.4 (4.8, 8.5)^a^	6.5 (5.2, 8.6)^a^	8.6 (6.5, 11.7)^b^	<0.001

Data are expressed as mean ± standard deviation or median (inter-quartile range).

SBP, systolic blood pressure; DBP, diastolic blood pressure; LDL, low-density lipoprotein; HDL, high-density lipoprotein; eGFR, estimated glomerular filtration rate; hsCRP, high-sensitivity C-reactive protein; CTRP-3, C1q/TNF-related Protein-3.

### Multiple logistic regression analysis for coronary artery disease as a dependent variable

We calculated odds ratio (OR) and 95% confidence interval (95% CI) using multiple logistic regression analysis for CAD as a dependent variable (Table 4). In an unadjusted model, subjects with the first (OR 3.46, 95% CI, 2.01-5.96) and second (OR 1.85, 95% CI, 1.08-3.18) tertile of CTRP-3 levels had significantly higher risk for CAD compared to those with the third tertile. Furthermore, after adjustment for age, gender, BMI, blood pressure, lipid profile, glucose, creatinine and hsCRP, the relationship between CTRP-3 levels and CAD were further intensified.

**Table 4:** Multiple logistic regression analysis for coronary artery disease as a dependent variable

	**First tertile**	**Second tertile**	**Third tertile**	***P***
Model 0	3.46 (2.01, 5.96)	1.85 (1.08, 3.18)	1	<0.001
Model 1	4.03 (2.24, 7.26)	2.13 (1.21, 3.75)	1	<0.001
Model 2	4.04 (2.24, 7.28)	2.53 (1.36, 4.70)	1	<0.001
Model 3	9.04 (2.81, 29.14)	5.14 (1.83, 14.42)	1	<0.001

Model 0, unadjusted.

Model 1, adjusted for age and gender.

Model 2, adjusted for age, gender and body mass index (BMI).

Model 3, adjusted for age, gender, BMI, systolic blood pressure, diastolic blood pressure, total cholesterol, LDL-cholesterol, HDL-cholesterol, triglyceride, glucose, creatinine and high sensitive C-reactive protein (hsCRP).

## Discussion

The present study first demonstrates that CTRP-3 concentrations are significantly decreased in patients with ACS or SAP compared to control subjects even after adjusting for other confounding factors. However, circulating progranulin levels were not significantly different between patients with CAD and control subjects.

Adiponectin is a representative adipokine that possess insulin sensitizing, anti-inflammatory, and anti-atherogenic properties [2]. Lower blood adiponectin levels are found in subjects with obesity, type 2 diabetes and metabolic syndrome. In a prospective cohort study for 3 years, we have shown that reduced adiponectin levels are independently associated with increased risk of both type 2 diabetes and metabolic syndrome [20]. Administration of adenovirus-mediated adiponectin reduces atherosclerotic lesion size in apolipoprotein E knockout mice [21]. Moreover, circulating adiponectin was shown to be lower in patients with CAD not only in cross-sectional studies [22], but also in prospective longitudinal studies [23]. However, some other studies did not found a value of adiponectin as a predictor of cardiovascular events. Two prospective studies found no significant association in either American Indians [24] or British women [25]. A meta-analysis has concluded that association between adiponectin and risk of coronary heart disease (CHD) is comparatively moderate, in contrast to the strong association between adiponectin and risk of type 2 diabetes [26]. Recently, Kim et al. demonstrated that neither adiponectin nor interleukin-6 predicted angiographic coronary artery disease in the presence of diabetes [27]. Furthermore, Baldasseroni et al. reported that adiponectin rises in patient with advanced heart failure, but this phenomenon is less evident in patients with type 2 diabetes [28]. The present study did not show significant difference of adiponectin levels between patients with ACS or SAP and control subjects, although adiponectin levels were negatively correlated with several cardiometabolic risk parameters reflecting obesity, dyslipidemia, hyperglycemia and inflammation.

Despite the wide range of beneficial effects of adiponectin, adiponectin-deficient animal models show a relatively modest phenotype unless metabolically challenged, whereas adiponectin transgenic mice show distinct improvements in the metabolic phenotypes. Therefore, this discrepancy suggests the existence of compensatory mechanisms that partially counteract the deficiency of adiponectin [29,30]. Recently, a novel family of adiponectin paralogs, designated C1q/TNF-related proteins (CTRPs), was discovered. Previous studies suggest the role of CTRP family proteins linking inflammation to metabolism [5]. CTRP-3 is expressed in human subcutaneous and visceral adipocytes, and plays an important role in adipocyte physiology [31]. CTRP-3 is a potent anti-inflammatory adipokine that inhibits pro-inflammatory signaling provoked by lipopolysaccharides (LPS), toll-like receptor (TLR) and fatty acids in adipocytes and monocytes [8]. CTRP-3 reduces tumor necrosis factor-α (TNF-α) and interleukin-6 (IL-6) secretion in LPS-treated monocytic cells through suppression of nuclear factor κB (NFκB) signaling pathway [32]. Interestingly, CTRP-3 directly stimulates adiponectin and resistin release, and serves as a regulator of adiponectin secretion in adipocytes [33]. In this study, circulating CTRP-3 levels showed a positive relationship with adiponectin and a negative relationship with hsCRP.

CTRP-3 has been reported to stimulate endothelial cell proliferation and migration in vitro [13]. Recently, Yi et al. demonstrated for the first time that expression and production of CTRP-3 are significantly reduced after MI and that administration of CTRP-3 improves survival and restores cardiac function in mice with coronary artery occlusion [14]. Therefore, they concluded that CTRP-3 is an adipokine possessing strong anti-remodeling and cardioprotective properties [14]. In agreement with the previous animal study, the present study showed significantly decreased CTRP-3 levels in patients with ACS or SAP compared to control subjects. Adjustment for cardiovascular risk factors further strengthens the relationship between CTRP-3 levels and CAD. Yi et al. suggested that preventing post-MI CTRP-3 inhibition or CTRP-3 supplementation may be a promising therapeutic approach for creating stable and functional vessels [14].

We previously identified progranulin as a novel marker of chronic inflammation in obesity and type 2 diabetes that reflect macrophage infiltration in omental adipose tissue [16]. Progranulin is recently re-discovered as an adipokine induced by TNF-α and dexamethasone [15]. Progranulin in blood and adipose tissue was dramatically increased in obese mouse models and progranulin deficient mice protected from high fat diet (HFD)-induced insulin resistance, adipocytes hypertrophy and obesity [15]. Pradeep et al. reported increased circulating progranulin and hsCRP levels in patients with obese subjects with chronic periodontitis, which is known as a risk factor for CAD [34]. In an immunohistochemical analysis of human carotid endarterectomy specimens, both macrophages and vascular smooth muscle cells in atherosclerotic plaque express progranulin [17]. Yilmaz et al. reported that patients with non-alcoholic fatty liver disease (NAFLD) have higher serum progranulin concentrations, which are positively associated with total cholesterol and LDL-cholesterol [35]. Recently, Qu et al. reported that plasma progranulin levels were higher in Chinese patients with type 2 diabetes and correlated with insulin resistance, inflammation and glycolipid metabolism [36]. In this study, progranulin concentrations showed modest negative relationship with HDL-cholesterol. Furthermore, patients with ACS or SAP did not show significant difference in progranulin levels compared to control subjects. Richter et al. reported that serum progranulin levels increase with deteriorating renal function [37]. However, adjusting for confounding factors including eGFR or creatinine levels did not affect the relationship between progranulin concentration and CAD in the present study. Previous studies suggest the complex dual role of progranulin that exerts anti-inflammatory and pro-inflammatory functions depending on target tissue. Tang et al. reported that progranulin binds directly to TNF receptors (TNFRs) and interfering the interaction between TNF-α and TNFR [38]. Full-length progranulin is generally anti-inflammatory, whereas proteolytically released granulins may have the opposite effect [39]. Further studies about the association between progranulin and atherosclerosis might be needed.

Some limitations of the study need to be considered. First, this is a cross-sectional analysis which restricts us to draw causal conclusions. Secondly, this study enrolled only Asian men and women. Therefore, the results of the present study may not apply to other ethnic populations.

## Conclusions

In conclusion, the present study clearly showed that patients with CAD (ACS or SAP) had significantly decreased circulating CTRP-3 concentration despite of similar progranulin and adiponectin levels after adjusting other cardiovascular risk factors. These results suggest the role of CTRP-3 linking obesity, inflammation and atherosclerosis.

## Competing interests

We declare the following interests. Byung-Soo Youn is employed by an AdipoGen, Inc. However, this does not alter our adherence to all the journal policies on sharing data and materials. No other potential conflicts of interest relevant to this article were reported.

## Authors’ contributions

KMC and SHS designed this study. KMC wrote the manuscript and researched data. SYH analyzed the data set. HCH, HYC and HJY collected data. BSY, SHB and HSS reviewed and edited the manuscript.

## References

[B1] Matsuzawa Y Nakamura T Shimomura I Kotani K Visceral fat accumulation and cardiovascular disease Obes Res 1995 3 Suppl 5 645S 647S 8653544 10.1002/j.1550-8528.1995.tb00481.x

[B2] Bluher M Clinical relevance of adipokines Diabetes Metab J 2012 36 5 317 327 10.4093/dmj.2012.36.5.317 23130315 PMC3486977

[B3] Barseghian A Gawande D Bajaj M Adiponectin and vulnerable atherosclerotic plaques J Am Coll Cardiol 2011 57 7 761 770 10.1016/j.jacc.2010.11.011 21310310

[B4] Wong GW Wang J Hug C Tsao TS Lodish HF A family of Acrp30/adiponectin structural and functional paralogs Proc Natl Acad Sci USA 2004 101 28 10302 10307 10.1073/pnas.0403760101 15231994 PMC478567

[B5] Schaffler A Buechler C CTRP family: linking immunity to metabolism Trends Endocrinol Metab 2012 23 4 194 204 10.1016/j.tem.2011.12.003 22261190

[B6] Seldin MM Tan SY Wong GW Metabolic function of the CTRP family of hormones Rev Endocr Metab Disord 2013 Epub ahead of print 10.1007/s11154-013-9255-7 PMC3931758 23963681

[B7] Hofmann C Chen N Obermeier F Paul G Buchler C Kopp A Falk W Schaffler A C1q/TNF-related protein-3 (CTRP-3) is secreted by visceral adipose tissue and exerts antiinflammatory and antifibrotic effects in primary human colonic fibroblasts Inflamm Bowel Dis 2011 17 12 2462 2471 10.1002/ibd.21647 21351204

[B8] Kopp A Bala M Buechler C Falk W Gross P Neumeier M Scholmerich J Schaffler A C1q/TNF-related protein-3 represents a novel and endogenous lipopolysaccharide antagonist of the adipose tissue Endocrinology 2010 151 11 5267 5278 10.1210/en.2010-0571 20739398

[B9] Peterson JM Wei Z Wong GW C1q/TNF-related protein-3 (CTRP3), a novel adipokine that regulates hepatic glucose output J Biol Chem 2010 285 51 39691 39701 10.1074/jbc.M110.180695 20952387 PMC3000950

[B10] Choi KM Hwang SY Hong HC Yang SJ Choi HY Yoo HJ Lee KW Nam MS Park YS Woo JT C1q/TNF-related protein-3 (CTRP-3) and pigment epithelium-derived factor (PEDF) concentrations in patients with type 2 diabetes and metabolic syndrome Diabetes 2012 61 11 2932 2936 10.2337/db12-0217 22837306 PMC3478553

[B11] Yoo HJ Hwang SY Hong HC Choi HY Yang SJ Choi DS Baik SH Bluher M Youn BS Choi KM Implication of progranulin and C1q/TNF-related protein-3 (CTRP3) on inflammation and atherosclerosis in subjects with or without metabolic syndrome PLoS One 2013 8 2 e55744 10.1371/journal.pone.0055744 23409033 PMC3567086

[B12] Li JM Zhang X Nelson PR Odgren PR Nelson JD Vasiliu C Park J Morris M Lian J Cutler BS Temporal evolution of gene expression in rat carotid artery following balloon angioplasty J Cell Biochem 2007 101 2 399 410 10.1002/jcb.21190 17171642

[B13] Maeda T Wakisaka S CTRP3/cartducin is induced by transforming growth factor-beta1 and promotes vascular smooth muscle cell proliferation Cell Biol Int 2010 34 3 261 266 10.1042/CBI20090043 19947921

[B14] Yi W Sun Y Yuan Y Lau WB Zheng Q Wang X Wang Y Shang X Gao E Koch WJ C1q/tumor necrosis factor-related protein-3, a newly identified adipokine, is a novel antiapoptotic, proangiogenic, and cardioprotective molecule in the ischemic mouse heart Circulation 2012 125 25 3159 3169 10.1161/CIRCULATIONAHA.112.099937 22653084 PMC3391311

[B15] Matsubara T Mita A Minami K Hosooka T Kitazawa S Takahashi K Tamori Y Yokoi N Watanabe M Matsuo E PGRN is a key adipokine mediating high fat diet-induced insulin resistance and obesity through IL-6 in adipose tissue Cell Metab 2012 15 1 38 50 10.1016/j.cmet.2011.12.002 22225875

[B16] Youn BS Bang SI Kloting N Park JW Lee N Oh JE Pi KB Lee TH Ruschke K Fasshauer M Serum progranulin concentrations may be associated with macrophage infiltration into omental adipose tissue Diabetes 2009 58 3 627 636 19056610 10.2337/db08-1147 PMC2646061

[B17] Kojima Y Ono K Inoue K Takagi Y Kikuta K Nishimura M Yoshida Y Nakashima Y Matsumae H Furukawa Y Progranulin expression in advanced human atherosclerotic plaque Atherosclerosis 2009 206 1 102 108 10.1016/j.atherosclerosis.2009.02.017 19321167

[B18] Thygesen K Alpert JS Jaffe AS Simoons ML Chaitman BR White HD Katus HA Lindahl B Morrow DA Joint ESCAAHAWHFTFftUDoMI Third universal definition of myocardial infarction Circulation 2012 126 16 2020 2035 10.1161/CIR.0b013e31826e1058 22923432

[B19] Levey AS Coresh J Greene T Stevens LA Zhang YL Hendriksen S Kusek JW Van Lente F Chronic kidney disease epidemiology C: using standardized serum creatinine values in the modification of diet in renal disease study equation for estimating glomerular filtration rate Ann Intern Med 2006 145 4 247 254 10.7326/0003-4819-145-4-200608150-00004 16908915

[B20] Choi KM Lee J Lee KW Seo JA Oh JH Kim SG Kim NH Choi DS Baik SH Serum adiponectin concentrations predict the developments of type 2 diabetes and the metabolic syndrome in elderly Koreans Clin Endocrinol (Oxf) 2004 61 1 75 80 10.1111/j.1365-2265.2004.02063.x 15212647

[B21] Okamoto Y Kihara S Ouchi N Nishida M Arita Y Kumada M Ohashi K Sakai N Shimomura I Kobayashi H Adiponectin reduces atherosclerosis in apolipoprotein E-deficient mice Circulation 2002 106 22 2767 2770 10.1161/01.CIR.0000042707.50032.19 12451000

[B22] Ouchi N Kihara S Arita Y Maeda K Kuriyama H Okamoto Y Hotta K Nishida M Takahashi M Nakamura T Novel modulator for endothelial adhesion molecules: adipocyte-derived plasma protein adiponectin Circulation 1999 100 25 2473 2476 10.1161/01.CIR.100.25.2473 10604883

[B23] Costacou T Zgibor JC Evans RW Otvos J Lopes-Virella MF Tracy RP Orchard TJ The prospective association between adiponectin and coronary artery disease among individuals with type 1 diabetes. The Pittsburgh epidemiology of diabetes complications study Diabetologia 2005 48 1 41 48 10.1007/s00125-004-1597-y 15616802

[B24] Lindsay RS Resnick HE Zhu J Tun ML Howard BV Zhang Y Yeh J Best LG Adiponectin and coronary heart disease: the strong heart study Arterioscler Thromb Vasc Biol 2005 25 3 e15 e16 10.1161/01.ATV.0000153090.21990.8c 15731480

[B25] Lawlor DA Davey Smith G Ebrahim S Thompson C Sattar N Plasma adiponectin levels are associated with insulin resistance, but do not predict future risk of coronary heart disease in women J Clin Endocrinol Metab 2005 90 10 5677 5683 10.1210/jc.2005-0825 16076942

[B26] Sattar N Wannamethee G Sarwar N Tchernova J Cherry L Wallace AM Danesh J Whincup PH Adiponectin and coronary heart disease: a prospective study and meta-analysis Circulation 2006 114 7 623 629 10.1161/CIRCULATIONAHA.106.618918 16894037

[B27] Kim JY Choi EY Mun HS Min PK Yoon YW Lee BK Hong BK Rim SJ Kwon HM Usefulness of metabolic syndrome score in the prediction of angiographic coronary artery disease severity according to the presence of diabetes mellitus: relation with inflammatory markers and adipokines Cardiovasc Diabetol 2013 12 140 10.1186/1475-2840-12-140 24088407 PMC3850730

[B28] Baldasseroni S Antenore A Di Serio C Orso F Lonetto G Bartoli N Foschini A Marella A Pratesi A Scarantino S Adiponectin, diabetes and ischemic heart failure: a challenging relationship Cardiovasc Diabetol 2012 11 151 10.1186/1475-2840-11-151 23249664 PMC3558365

[B29] Davis KE Scherer PE Adiponectin: no longer the lone soul in the fight against insulin resistance? Biochem J 2008 416 2 e7 e9 10.1042/BJ20082033 18990088

[B30] Wong GW Krawczyk SA Kitidis-Mitrokostas C Revett T Gimeno R Lodish HF Molecular, biochemical and functional characterizations of C1q/TNF family members: adipose-tissue-selective expression patterns, regulation by PPAR-gamma agonist, cysteine-mediated oligomerizations, combinatorial associations and metabolic functions Biochem J 2008 416 2 161 177 10.1042/BJ20081240 18783346 PMC3936483

[B31] Schmid A Kopp A Hanses F Bala M Muller M Schaffler A The novel adipokine C1q/TNF-related protein-3 is expressed in human adipocytes and regulated by metabolic and infection-related parameters Exp Clin Endocrinol Diabetes 2012 120 10 611 617 23174996 10.1055/s-0032-1323803

[B32] Weigert J Neumeier M Schaffler A Fleck M Scholmerich J Schutz C Buechler C The adiponectin paralog CORS-26 has anti-inflammatory properties and is produced by human monocytic cells FEBS Lett 2005 579 25 5565 5570 10.1016/j.febslet.2005.09.022 16213490

[B33] Wolfing B Buechler C Weigert J Neumeier M Aslanidis C Schoelmerich J Schaffler A Effects of the new C1q/TNF-related protein (CTRP-3) “cartonectin” on the adipocytic secretion of adipokines Obesity (Silver Spring) 2008 16 7 1481 1486 10.1038/oby.2008.206 18421280

[B34] Pradeep AR Priyanka N Prasad MV Kalra N Kumari M Association of progranulin and high sensitivity CRP concentrations in gingival crevicular fluid and serum in chronic periodontitis subjects with and without obesity Dis Markers 2012 33 4 207 213 10.1155/2012/173123 22960346 PMC3810772

[B35] Yilmaz Y Eren F Yonal O Polat Z Bacha M Kurt R Ozturk O Avsar E Serum progranulin as an independent marker of liver fibrosis in patients with biopsy-proven nonalcoholic fatty liver disease Dis Markers 2011 31 4 205 210 10.1155/2011/126240 22045426 PMC3826876

[B36] Qu H Deng H Hu Z Plasma progranulin concentrations are increased in patients with type 2 diabetes and obesity and correlated with insulin resistance Mediators Inflamm 2013 2013 360190 23476101 10.1155/2013/360190 PMC3588183

[B37] Richter J Focke D Ebert T Kovacs P Bachmann A Lossner U Kralisch S Kratzsch J Beige J Anders M Serum levels of the adipokine progranulin depend on renal function Diabetes Care 2013 36 2 410 414 10.2337/dc12-0220 23033238 PMC3554312

[B38] Tang W Lu Y Tian QY Zhang Y Guo FJ Liu GY Syed NM Lai Y Lin EA Kong L The growth factor progranulin binds to TNF receptors and is therapeutic against inflammatory arthritis in mice Science 2011 332 6028 478 484 10.1126/science.1199214 21393509 PMC3104397

[B39] Cenik B Sephton CF Kutluk Cenik B Herz J Yu G Progranulin: a proteolytically processed protein at the crossroads of inflammation and neurodegeneration J Biol Chem 2012 287 39 32298 32306 10.1074/jbc.R112.399170 22859297 PMC3463300

